# Impact of health literacy on pregnancy outcomes in socioeconomically disadvantaged and ethnic minority populations: A scoping review

**DOI:** 10.1002/ijgo.15852

**Published:** 2024-08-22

**Authors:** Jiwon Kim, Alexander E. P. Heazell, Maya Whittaker, Tomasina Stacey, Kylie Watson

**Affiliations:** ^1^ Division of Developmental Biology and Medicine, Maternal and Fetal Health Research Centre, School of Medical Sciences, Faculty of Biology, Medicine and Health University of Manchester Manchester UK; ^2^ Saint Mary's Hospital Manchester University NHS Foundation Trust Manchester UK; ^3^ Faculty of Nursing Midwifery and Palliative Care King's College London London UK; ^4^ Division of Nursing, Midwifery and Social Work, School of Health Sciences, Faculty of Biology, Medicine and Health University of Manchester Manchester UK

**Keywords:** health literacy, maternal death, minority population, perinatal death, pregnancy, socioeconomic status, stillbirth

## Abstract

**Background:**

Health literacy, influenced by sociodemographic characteristics such as ethnicity, economic means and societal factors, affects the ways in which pregnant women maintain their health; this in turn may increase risk of adverse pregnancy outcomes.

**Objective:**

To explore what is known about the impact of personal health literacy on prevention of stillbirth and related adverse outcomes in pregnant women of low socioeconomic status or from ethnic minority backgrounds.

**Search Strategy:**

MEDLINE, CINAHL, PsychINFO, and CENTRAL were searched as well as reference lists of included studies and gray literature.

**Selection Criteria:**

Included studies focused on personal health literacy and stillbirth prevention in women from low socioeconomic or ethnic minority backgrounds in the perinatal period.

**Data Collection and Analysis:**

A meta‐summary approach was adopted for qualitative, observational, descriptive, and audit studies. Findings of intervention studies were extracted, and meta‐analyses were conducted where possible. The primary outcome was stillbirth; maternal mortality and neonatal mortality were secondary outcomes.

**Main Results:**

Forty‐one studies were included from diverse geographical settings. The meta‐summary synthesized five abstracted statements. These recognized lower personal health literacy and greater difficulty interacting with healthcare services in the studied populations, primarily as the result of limited health knowledge, lack of positive perception towards health services, language barriers, illiteracy, and relying on friends or family members for health information. Meta‐analysis of intervention studies revealed no association between current interventions that aimed to increase personal health literacy and the risk of stillbirth (relative risk [RR] 1.04, 95% confidence interval [CI] 0.96–1.12), neonatal mortality (RR 0.88, 95% CI 0.75–1.03), and maternal mortality (RR 0.87, 95% CI 0.63–1.22).

**Conclusions:**

Various factors suggest lower personal health literacy in women of low socioeconomic status or ethnic minority, which can increase the risk of stillbirth. However, this review identified no significant impact of current health education interventions on the risk of stillbirth, or neonatal or maternal mortality. Although not directly measured, the health education interventions were anticipated to increase personal health literacy. Further research on the topic of this scoping review is warranted, particularly in lower‐resource settings and regarding the potential role of e‐literacy and organizational health literacy to improve pregnancy outcomes. To address deficits in health literacy, efforts must be made to provide pregnant women with health information in novel, accessible ways.

## Synopsis

Current health educational interventions, improving low health literacy in socioeconomically deprived and ethnic minority women, do not significantly reduce stillbirth, or maternal and neonatal mortality.

## 1 INTRODUCTION

Approximately 1.9 million stillbirths occurred globally in 2021, making this adverse pregnancy outcome a significant health burden.^1^ Despite the 47.0% reduction in stillbirth to 13.9 per 1000 births from 1990 to 2015,^2^ the rate exceeds the target set by the Every Newborn Action Plan of 12 per 1000 by 2020.^3^ Certain groups have consistently had a higher stillbirth risk, including ethnic minorities,^4^ migrant populations,^5^ and those with low socioeconomic status.^6^ For example, Black African, Black Caribbean, and Pakistani women in the UK have a higher stillbirth rate compared with White women.^4^ Similarly, an association between socioeconomic deprivation and stillbirth exists.^6^, ^7^ The complex mechanisms underpinning these inequalities in stillbirth rate range from inability to afford perinatal care,^8^ late booking and infrequent attendance of prenatal care,^9^ certain sociocultural norms,^10^ reduced autonomy in healthcare‐related decision making,^11^ and racism.^12^

Insufficient personal health literacy may be one mechanism by which women from socioeconomically deprived or ethnic minority backgrounds experience worse health outcomes. Health literacy describes the ability to access and interact with available health information to make decisions about maintaining one's health.^13^, ^14^, ^15^, ^16^ Studies have demonstrated that people from ethnic minority groups are more likely to have lower health literacy.^13^, ^17^, ^18^ Insufficient health literacy is also associated with socioeconomic factors including immigration, low levels of income, and low educational attainment.^15^ Reduced ability to understand and appraise health information and communicate with health professionals may lead to suboptimal health.^16^ Engagement with health services during pregnancy is important for fetal and maternal health.^19^, ^20^ Although organizations have a crucial role in facilitating individuals' interactions with health information and services, this review focuses on personal health literacy.^21^

As social inequalities are associated with poor health literacy and stillbirth, exploring the possible interaction between health literacy in women from ethnic minority or low socioeconomic backgrounds and stillbirth prevention is important. A preliminary search for existing scoping reviews on this topic was conducted in April 2023 on Medline, Cochrane, CINAHL, and PsycINFO, which yielded no results. This study was undertaken to address this research gap.

This scoping review sought to assess and analyze the key messages present in current literature on the role of personal health literacy in stillbirth prevention in socioeconomically disadvantaged pregnant women or those from ethnic minority groups. Additionally, it aimed to assess the effects of health education interventions, addressing health literacy, on stillbirth, and neonatal and maternal mortality.

## 2 MATERIALS AND METHODS

An a priori protocol, detailing the criteria for including and excluding reports was developed and published online before the search process.^22^ The Arksey and O'Malley^23^ scoping review framework was used to assess the extent of available research and identify knowledge gaps. The PRISMA‐ScR checklist was followed for conduct and reporting of this scoping review (Table S1). Before searching, key terms were defined, as follows: stillbirth is the death of the fetus before or during labor (although there is international variation on nomenclature depending on fetal weight and gestational age, this review included studies irrespective of the local definition^24^). The main components of personal health literacy according to Liu et al.^16^ are: “a. knowledge of health, healthcare and health systems; b. processing and using information in various formats in relation to health and healthcare; and c. ability to maintain health through self‐management and working in partnerships with health providers.” Ethnic minority groups are defined as those, in which members share culture, language, history or tradition, and that make up less than 50% of the population of the territory they inhabit,^25^ and include migrants. Individuals with low socioeconomic status are those who have reduced access to financial, social, educational, and health resources.^26^

### 2.1 Information sources and search strategy

Medline, Cochrane Central Register of Clinical Trials, CINAHL, and PsychINFO were searched at the end of April 2023, following publication of the protocol. LILACS was searched in July 2024, during the editing process of the manuscript. Search strategies were developed using synonyms and subject headings relevant to the terms “stillbirth,” “health literacy,” “low socioeconomic status,” and “ethnic minority.” Boolean operators were used. Table S2 shows the search strategies. The results from each database were exported to Covidence (Melbourne, Australia) where duplicates were removed.

### 2.2 Eligibility criteria and study selection

Studies meeting the following criteria were included: (1) reports including women in the perinatal period, from conception to birth or women who have experienced stillbirth; (2) the manuscript focused on stillbirth prevention; reports discussing different forms of perinatal deaths were included only when extraction of data regarding stillbirth was possible (review of manuscripts revealed some studies irrelevance to the scoping review as they only mentioned stillbirth history; therefore, this criterion was amended from the protocol to direct the focus onto stillbirth prevention); (3) there was a clear description of concepts regarding personal health literacy, as defined in the Materials and methods section above, in the title or the abstract; and (4) the manuscript focused on women of low socioeconomic status or from ethnic minority backgrounds as defined above. Reports were not excluded based on language, the publication year, or the assessment of quality as per guidance for scoping reviews.

Two investigators screened the titles and abstracts (JK and MW). Two additional investigators (AH and KW) resolved any disagreements. One investigator screened the full texts of the agreed sources using the eligibility criteria. Systematic reviews, literature reviews, and meta‐analyses fitting the inclusion criteria were used to identify sources, and the selection criteria were used by two authors (JK and AH) to extract and screen individual sources.

### 2.3 Manual search

Gray literature databases (DART‐Europe E‐theses Portal, DANS EASY Archive, and EThOS) were manually searched. To retrieve studies, the titles and abstracts of the papers appearing with the term “stillbirth” were screened using the eligibility criteria, followed by a full‐text screen. African Journals Online was manually searched similarly but “‘health literacy’ pregnancy outcomes” was used instead to retrieve studies. Additional sources were identified from reference lists of all included studies.

### 2.4 Data extraction

For each study, the following key information was recorded: author, title, publication year, context, country setting, sample size, study type, ethnicity or socioeconomic factor, health literacy concept (i.e., knowledge and understanding of pregnancy‐related information; knowledge and perception of health service; obtaining information; form of literacy; language barrier; and compliance), and findings addressing the objective of this study. The countries' income levels were determined using the World Bank's classification system.^27^

### 2.5 Assessment of risk of bias

Due to the nature of a scoping review, quality assessment and assessment of risk of bias for included studies were not conducted.^28^

### 2.6 Data analysis and synthesis

To synthesize the non‐intervention studies' findings, the meta‐summary technique of Sandelowski et al.^29^ was adopted. Data from each report were extracted, then grouped together based on similarity. These groups were used for constructing abstracted statements that concisely, but comprehensively, synthesize each group's findings. Frequency effect size (FES) measuring how frequently an abstracted statement appeared in the included studies was calculated using the following equation.

$$\mathrm{FES}=\left(\text{number of reports containing}\;a\;\text{finding}/\text{total number of reports}\right).$$

A meta‐analysis was conducted for intervention studies. Interventions used a form of health education, one way of increasing personal health literacy, with stillbirth, neonatal mortality, and maternal mortality as outcomes. Following data extraction, data were tabulated and meta‐analysis was performed using the *metan* and *metafunnel* commands in STATA 14 (StataCorp, College Station, TX, USA). Forest plots were used to evaluate the association between the interventions and the risk of adverse pregnancy outcomes, and funnel plots visualized the risk of publication bias. Heterogeneity was classified using the thresholds for *I* ^2^ outlined in the Cochrane Handbook; heterogeneity was potentially unimportant if 0%–40%, moderate if 30%–60%, substantial if 50%–90% and considerable if 75%–100%.^30^

## 3 RESULTS

### 3.1 Study selection

**FIGURE 1:**
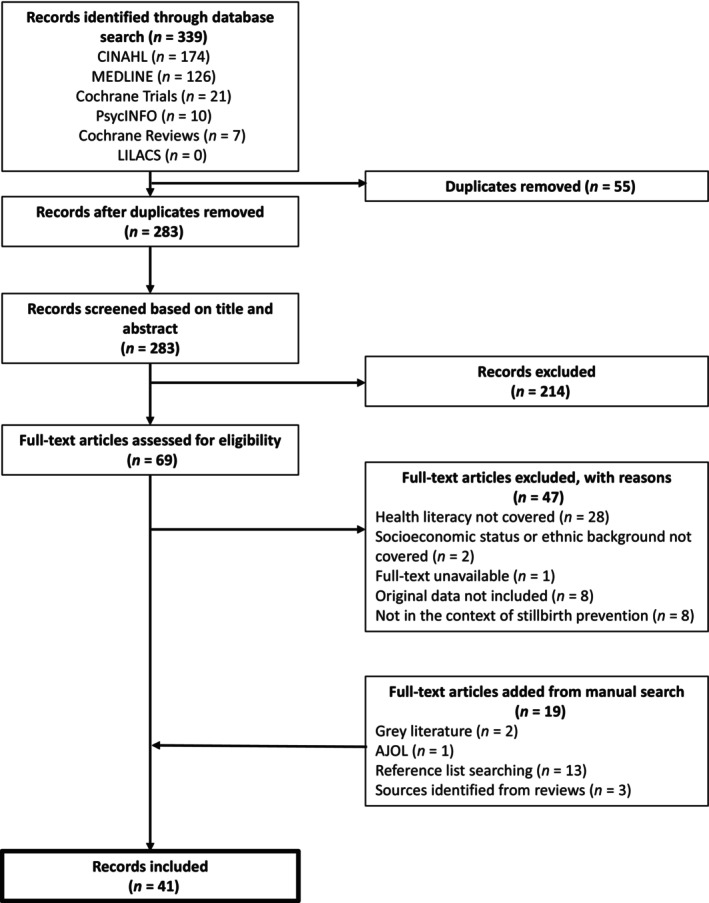
PRISMA flow diagram.

Initial searches identified 339 reports. Of these, 55 duplicates were removed (Figure 1). After title and abstract screening, full‐text screening, and addition of reports from manual searching, 41 reports, published between 2000 and 2024, were included in this review. One report was in French^31^ and the rest were in English.

### 3.2 Study characteristics

**FIGURE 2:**
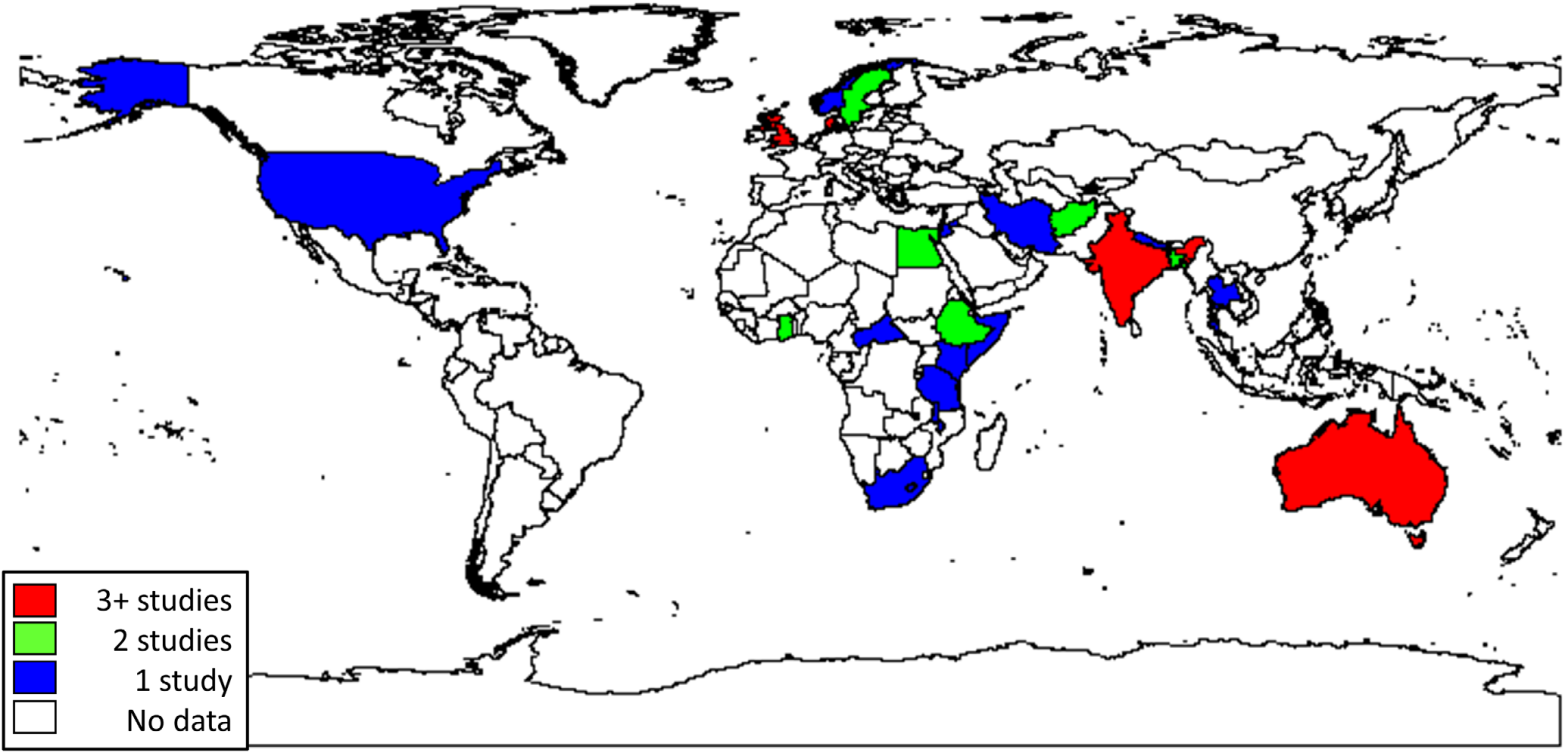
Geographical distribution of included studies.

The reports were grouped by study design: qualitative (*n* = 12), descriptive survey (*n* = 9), observational analytic (*n* = 8), trial (*n* = 10), and perinatal audit (*n* = 2) (Tables S3–S7, respectively). Most studies were conducted in high‐income countries (*n* = 17), followed by lower‐middle‐income (*n* = 14), low‐income (*n* = 7), and upper‐middle‐income (*n* = 3) countries. Figure 2 shows the geographical distribution of studies; South Asia had the greatest concentration of studies. South America was the only continent with no studies included in the review.

### 3.3 Meta‐summary

The meta‐summary of qualitative and quantitative data from the non‐intervention studies resulted in five abstracted statements. The FES of each statement ranged between 19.4% and 45.2%.

Knowledge and awareness of stillbirth, the risk factors, and the danger signs in pregnancy were limited in women of low socioeconomic status or from ethnic minority background^11^, ^14^, ^32^, ^33^, ^34^, ^35^, ^36^, ^37^, ^38^, ^39^, ^40^, ^41^, ^42^, ^43^ (FES 45.2%).

Women of low socioeconomic status or those from an ethnic minority background lacked general knowledge regarding safe pregnancy and stillbirth prevention.^14^, ^32^, ^34^, ^35^, ^36^, ^39^, ^42^ Recognition and awareness of birth complications and their severity were limited.^32^, ^38^, ^41^ There was low awareness of risk factors for adverse pregnancy outcomes, such as syphilis infection^43^ or engagement in strenuous physical work or medications that could be harmful to the fetus.^11^ Furthermore, awareness of optimal birth spacing,^11^ safe physical exercise,^33^ quality diet,^40^ and the need for glycemic control in women with gestational diabetes mellitus was limited.^37^

There was a lack of positive perception of and familiarity with healthcare services among women of low socioeconomic status or from ethnic minority backgrounds^8^, ^11^, ^31^, ^32^, ^39^, ^42^, ^44^, ^45^, ^46^ (FES 29.0%).

Women lacked a comprehensive knowledge of available healthcare services,^32^, ^45^, ^46^ including their locations^8^ and affordability,^44^ and were unfamiliar with referral systems.^42^ Prenatal care was commonly perceived as unnecessary.^11^, ^31^ Recommended surgical interventions such as cesarean section were often declined because of concerns about negative health impacts, including infertility.^11^ Some participants held misinformation about maternity services, believing that the fetal anomaly scan would harm the fetus.^39^

Migrant women often faced language barriers when interacting with healthcare services, resulting in miscommunication^8^, ^32^, ^40^, ^47^, ^48^, ^49^, ^50^, ^51^, ^52^, ^53^ (FES 32.3%).

Not speaking the local language was considered a barrier^32^ as it was associated with difficulty understanding the requirements for a healthy pregnancy,^40^ delayed perinatal care attendance,^8^ and even an increased risk of stillbirth.^49^, ^51^ Professional translation services were helpful^48^; but were not always provided,^50^, ^52^ and sometimes led to miscommunication and poor conveyance of important information regarding a healthy pregnancy.^52^, ^53^ Reducing this barrier through health education materials available in various languages facilitated accessing information and increased understanding.^47^

Women sought out pregnancy‐related information mainly from their friends or family, replacing “official” sources.^8^, ^14^, ^32^, ^33^, ^34^, ^45^, ^54^ This included information on prenatal care,^8^ specific illnesses,^34^ and lifestyle advice.^14^, ^33^ There was often an inconsistency between the information given by healthcare professionals and that provided by external sources, leading to confusion,^14^ Women used the internet to obtain information^14^ for its perceived accessibility.^45^ Overall, there was variation in the perceived trustworthiness of external sources such as friends, family members, or the internet.^45^, ^54^

Increased rates of illiteracy, often seen in women of low socioeconomic or ethnic minority background, were associated with difficulty with engaging with healthcare services as well as increased stillbirth risk^14^, ^31^, ^40^, ^55^, ^56^, ^57^ (FES 19.4%).

Many women in these groups had low literacy,^14^ primarily due to low education,^56^ and experienced greater risk for stillbirth.^55^, ^56^, ^57^ Difficulty understanding written information often became a barrier to receiving information from healthcare professionals^40^ and attending prenatal care.^31^

### 3.4 Meta‐analysis of trials

There were 10 intervention studies,^58^, ^59^, ^60^, ^61^, ^62^, ^63^, ^64^, ^65^, ^66^, ^67^ nine were cluster randomized controlled trials and one was an interventional evaluation study.^67^ Of the 10 health education interventions, seven involved community‐based participatory women's groups^58^, ^60^, ^61^, ^62^, ^63^, ^65^, ^66^ in which women obtained health knowledge in a proactive, engaging manner through phases of discussions and developed strategies for identified problems. Two reports were based on the same intervention in Denmark involving information leaflets and a multilingual smartphone app with midwives trained in intercultural communication.^59^, ^64^ One study involved teaching and distribution of educational materials.^67^ Stillbirth and neonatal mortality were measured in eight studies^58^, ^59^, ^60^, ^61^, ^62^, ^63^, ^65^, ^66^ and maternal mortality in seven studies.^58^, ^60^, ^61^, ^62^, ^63^, ^65^, ^66^ The definition of stillbirth was inconsistent across the eight studies, with a lack of clear definition in four trials.^60^, ^62^, ^65^, ^66^ The small numbers of studies meant that a subgroup analysis could not be conducted based upon the gestational age definition of stillbirth.

**FIGURE 3:**
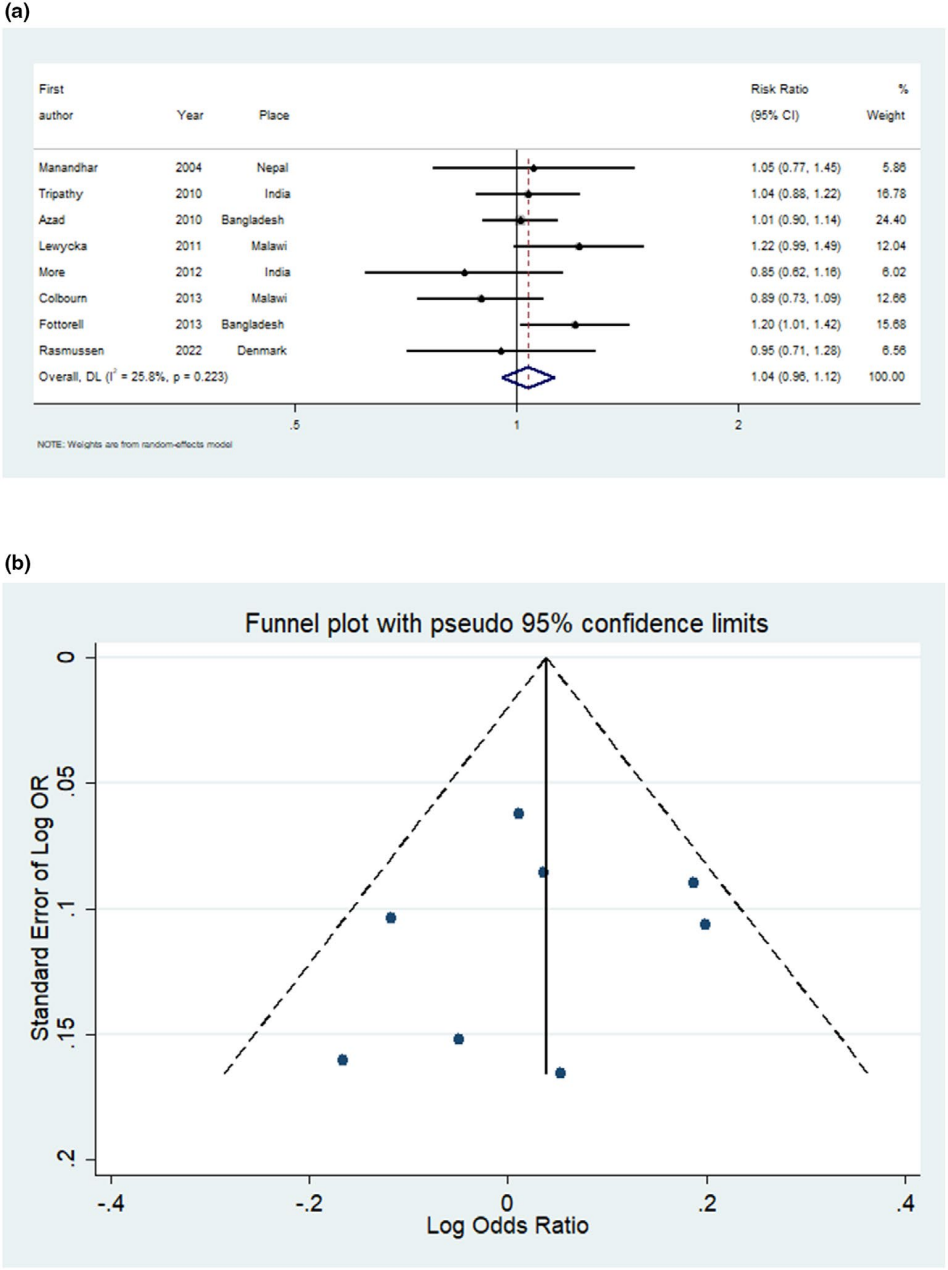
(a) Forest plot for meta‐analysis of studies measuring risk ratio of stillbirth after exposure to participatory womens group intervention. (b) Funnel plot of the studies measuring stillbirth outcome after exposure to participatory womens group intervention.

When participants were exposed to the interventions there was no change in stillbirth (relative risk [RR] 1.04, 95% confidence interval [CI] 0.96–1.12, Figure 3a, eight studies 177 255 participants). There was a potentially unimportant heterogeneity of 25.8%. The study by Azad et al.^63^ contributed the most to the pooled result, accounting for 24.4%.

**FIGURE 4:**
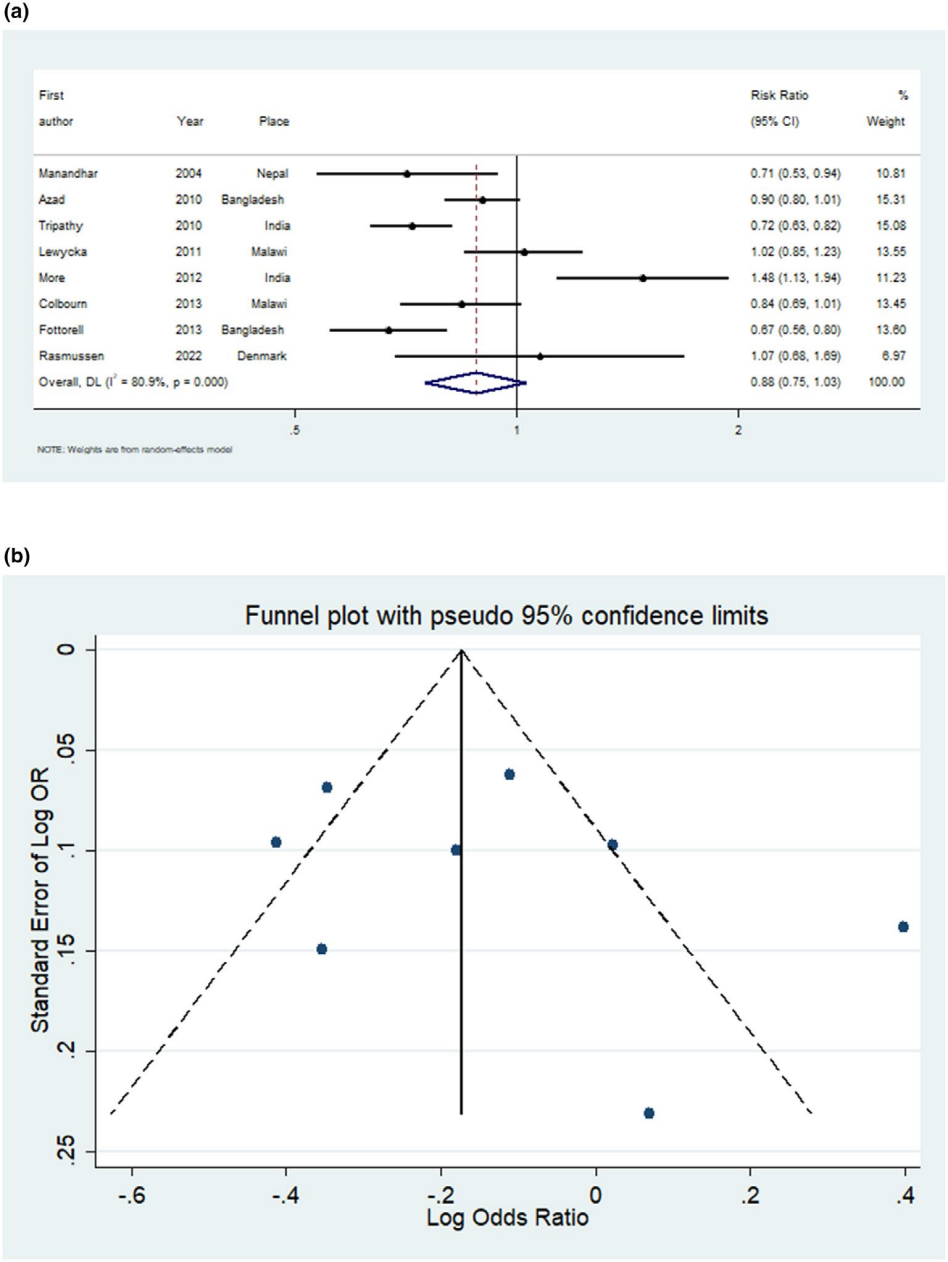
(a) Forest plot for meta‐analysis of studies measuring risk ratio of neonatal mortality after exposure to participatory womens group intervention. (b) Funnel plot of the studies measuring neonatal mortality after exposure to participatory womens group intervention.

Exposure to the interventions resulted in a statistically non‐significant 12% reduction in neonatal death, which approached statistical significance (RR 0.88, 95% CI 0.75–1.03, Figure 4a, eight studies 177 255 participants). However, heterogeneity was substantial or considerable (80.9%, Figure 4a). The studies by Tripathy et al.^62^ and Azad et al.^63^ had the greatest weights, of 15.1% and 15.3%, respectively, and both showed a reduction in neonatal mortality.

**FIGURE 5:**
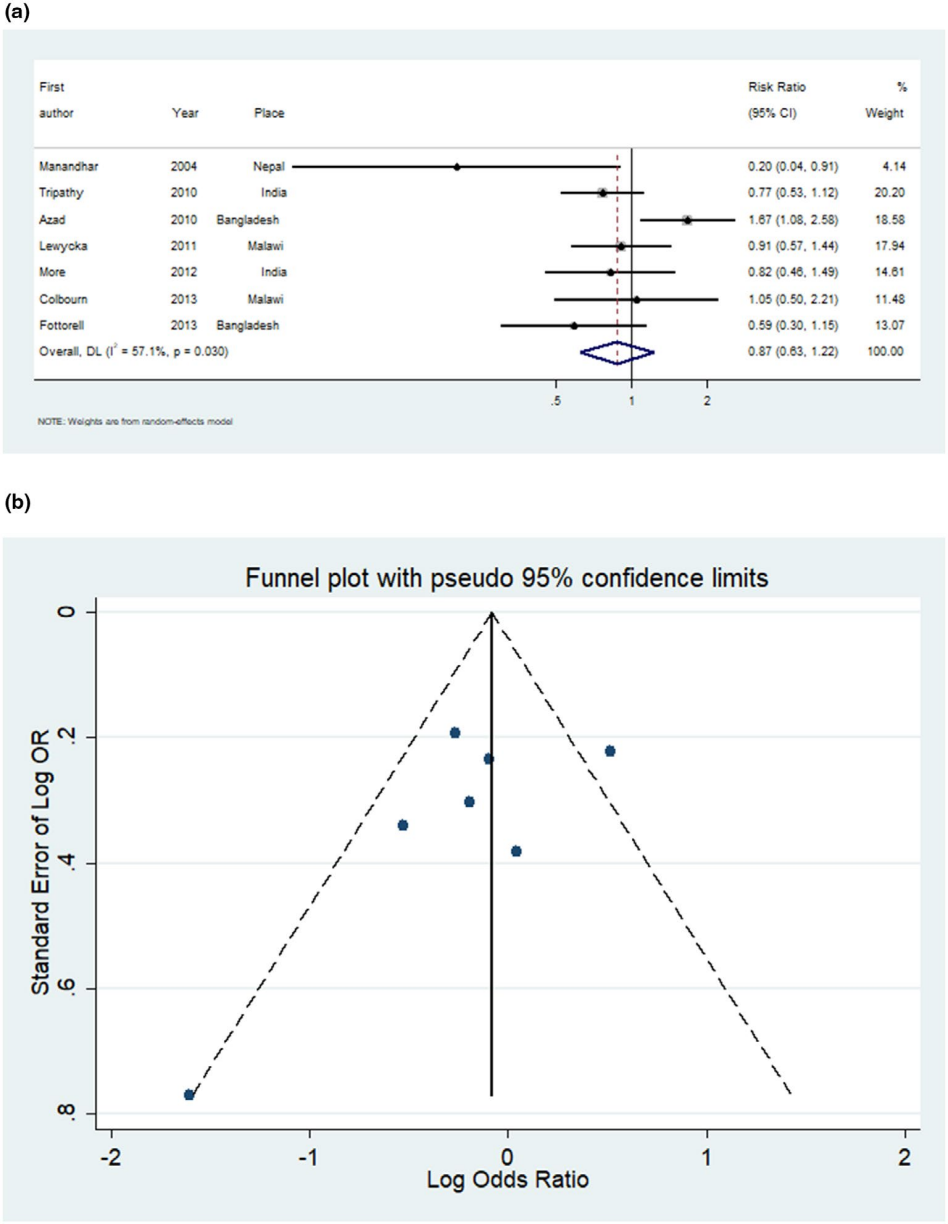
(a) Forest plot for meta‐analysis of studies measuring risk ratio of maternal mortality after exposure to participatory womens group intervention. (b) Funnel plot of the studies measuring maternal mortality after exposure to participatory womens group intervention.

Health education interventions did not reduce maternal mortality (RR 0.87, 95% CI 0.63–1.22, Figure 5a, seven studies 126 082 participants). However, heterogeneity was substantial (63.8%).

The funnel plots for the impact of the interventions on stillbirth, neonatal mortality, and maternal mortality showed no evidence of asymmetry, indicating low risks of publication bias (Figures 3, 4, 5).

The meta‐analysis omitted two studies,^64^, ^67^ which measured different outcomes. The intervention evaluation study by Metwally et al.^67^ demonstrated a significant increase in prenatal care uptake and a decrease in perinatal complications including stillbirth, low birth weight, and birth defects, after exposure to teaching and educational materials on pregnancy‐related information. Rasmussen et al.^64^ however, demonstrated that their intervention did not improve non‐Western immigrant women's health literacy, measured by their engagement with and ability to navigate health services, and awareness of complication management.

## 4 DISCUSSION

This scoping review synthesized data from 41 reports addressing the role of personal health literacy in the prevention of stillbirth and related adverse perinatal outcomes in women of low socioeconomic status or from ethnic minority background. The meta‐summary demonstrated that reduced personal health literacy could be attributable to multiple factors, some of which may be amenable to intervention. However, the meta‐analysis revealed that current health education interventions did not significantly reduce stillbirth, maternal mortality, and neonatal mortality. The substantial heterogeneity in the effect of the interventions does not appear to be related to geographic location or year of studies.^58^, ^62^

The types of health literacy concepts identified in reports within qualitative, descriptive survey, observational analytic and perinatal audit study designs were: knowledge of pregnancy‐related information; knowledge and perception of health services; obtaining information; literacy; language barriers; and adherence. The health literacy concepts discussed in intervention reports were grouped under “health education.” Qualitative reports addressed all identified health literacy concepts. On average, each qualitative report addressed two concepts, whereas each report of the rest of the study designs usually addressed a single concept. Out of the 12 qualitative studies, eight were conducted in high‐income countries,^14^, ^32^, ^33^, ^39^, ^40^, ^45^, ^47^, ^54^ suggesting a limited insight into the given topic in low‐resource, higher‐burden countries.

Twenty studies were from higher‐income (high‐ or upper‐middle‐income) countries and 21 were from lower‐income (low‐ or lower‐middle‐income) countries. However, this geographical distribution does not reflect the geographical distribution of stillbirths (or related deaths) as most stillbirths (and neonatal/maternal deaths) happen in low‐ and middle‐income countries, demonstrated by a third of the total number of stillbirths in 2019 occurring in India, Pakistan, and Nigeria alone.^68^ Hence, research into health literacy for perinatal and maternal health is urgently required in lower‐income, high‐burden countries.

The five abstracted statements of the meta‐summary found limited knowledge of stillbirth‐related information in the studied populations, though it should be acknowledged that there was variation between studies. For example, in the study conducted in Jordan,^35^ women's knowledge regarding toxoplasmosis, a risk factor for adverse pregnancy outcome, was associated with ethnicity but not with income or employment status. However, this lack of association between socioeconomic status and health literacy is an uncommon finding, with the opposite being established in current literature.^13^, ^15^ Although the study by Creanga et al.^69^ conducted in Kenya, demonstrated participants strong intention to use healthcare services during pregnancy and birth, the meta‐summary identified many participants unfamiliarity with the availability and importance of maternity services. Hence, a desire to engage with health services may not directly translate into use of maternity services if practical barriers are not addressed.

Language barrier was a common experience among migrant women as they often faced difficulty communicating with health services. The UK National Institute for Health and Care Excellence guidelines^70^ recommend providing interpretation services to non‐English speaking women. However, use of professional interpreters remains low,^71^ and has been identified as a contributory issue in Confidental Enquiries into Maternal Death and Stillbirth in the UK.^71^ Therefore, increased efforts must be made to ensure that the necessary interpreting services are consistently offered, and that health promotion tools, e.g. videos and literature, are in appropriate languages.

Women's primary sources of information included friends, family, and the internet. Despite recognizing the potential unreliability of such sources,^14^, ^45^ their concerns regarding financial barriers to care,^11^ mistrust in healthcare providers,^72^ unfamiliarity with services,^11^, ^31^, ^46^ and language barriers^32^, ^40^, ^50^ could influence them to seek medical advice from more easily accessible sources rather than established, reliable sources.

Finally, illiteracy was recognized to have a major role, and sometimes, a lack of educational attainment was labeled as illiteracy.^31^, ^55^, ^56^ Reading, writing, and numerical literacies are essential for interacting with health information, and are skills developed at educational institutions.^73^ Therefore, the association between illiteracy and increased risk of stillbirth is plausible and requires targeted efforts to provide accessible health promotion tools.

There were similar numbers of studies conducted regarding knowledge of stillbirth‐related information, familiarity with health services, and literacy in both higher‐ and lower‐income countries, suggesting that these phenomena are pertinent, irrespective of a country's income level. However, data on the role of language barriers was only extracted from studies in higher‐income countries.^8^, ^32^, ^40^, ^47^, ^48^, ^49^, ^50^, ^51^, ^52^, ^53^ Similarly, among the studies reporting on women seeking medical advice from external sources, six out of seven were from higher‐income settings.^8^, ^14^, ^32^, ^33^, ^45^, ^54^ This imbalance demonstrates that language barriers and obtaining information from non‐official sources occur more commonly in higher‐income countries. An overwhelming majority of the studies^14^, ^32^, ^40^, ^45^, ^47^, ^48^, ^49^, ^50^, ^51^, ^52^, ^53^, ^54^ included in those abstracted statements focused on migrant women. This suggests that such challenges are frequently faced by migrant women in higher‐income countries who may not speak the local language, leading to a reliance on familiar information sources such as family and friends.

The meta‐analysis demonstrated that exposure to health education interventions, and therefore an anticipated increase in personal health literacy, did not result in a significant change in stillbirth, maternal mortality, or neonatal mortality. Although the exact reasons for the lack of effect is unclear, participatory women's groups being an intervention in seven out of the eight intervention studies could explain the phenomenon. Women's groups encourage active discussion and involvement. Information is shared in a culturally appropriate context, and the members identify problem areas and strategize to minimize those issues, mainly focusing on maternal and neonatal health problems.^60^, ^61^, ^62^, ^63^, ^65^ However, this interactive nature allows the contents of the discussions to be flexible to the priorities of the group. If the participating women have low awareness of adverse birth outcomes or these topics are stigmatized, then discussions and active planning regarding prevention might be inadequate, despite the facilitator's previous training and planning. Poor recognition of adverse perinatal outcomes has been observed in women from slum settlements in India,^38^ and stillbirth is commonly stigmatized.^74^ However, the initial focus on maternal and neonatal mortality could explain the non‐statistically significant trend towards a reduction in maternal (RR 0.87, 95% CI 0.63–1.22) and neonatal (RR 0.88, 95% CI 0.75–1.03) deaths. This trend may also reflect the inclusion of reducing maternal and infant mortality in the Sustainable Development Goals, whereas stillbirth was omitted.^75^ Therefore, countries with the highest burden of maternal and perinatal mortality may have focused health literacy on signs and symptoms of maternal disease or newborn survival rather than on fetal well‐being; this is supported by the first theme of the meta‐synthesis, which identified poor knowledge of warning signs for stillbirth among participants.

Co‐creation could potentially improve the effectiveness of health education interventions. Only the MAMAACT intervention of Rasmussen et al.^59^ was co‐created, in which stakeholders,^76^ such as midwives and a non‐profit organization associated with immigrant women, were actively involved in the development of the intervention. Co‐creation allows participation of those affected by the intervention who have instrumental knowledge and skills, resulting in an intervention that can be more implementable, sustainable, and effective^76^, ^77^ Therefore, with co‐creation, intervention studies could develop appropriate interventions which may reduce adverse perinatal outcomes, although this was not reflected in the Rasmussen et al. study.^59^, ^78^ True co‐creation approaches require investment and further evaluation.

This scoping review was strengthened by an inclusive search process; searching gray literature databases and reference lists and favoring inclusion over exclusion allowed the inclusion of a diverse range of studies with no limitation on publication year, language, or study design. Using a meta‐analysis for intervention studies and a meta‐summary for descriptive studies prevented an overgeneralization of the findings. Different definitions of stillbirth were accepted in this study to acknowledge the variations between settings.

One limitation of this review is the potential presence of bias during the screening and data extraction. Two reviewers conducted the title and abstract screening, and two additional investigators resolved disagreements. However, the full‐text screening, the manual searching, and the data extraction process were conducted by one investigator because of time and resource constraints. Therefore, the final inclusion of the studies and extraction of data were based on the primary investigator's judgments, rendering them vulnerable to potential bias.

Some valuable findings were not reported in the meta‐summary, as they did not appear frequently enough to be grouped under an abstracted statement. For instance, e‐illiteracy and its impact on women's ability to interact with online health information were mentioned in two studies.^14^, ^47^ However, as it did not appear in other studies, it did not have sufficient weight to be considered an abstracted statement. This insufficient weight of the topic of e‐literacy suggests that further research investigating its link to stillbirth prevention is required, especially in higher‐income countries where technology such as smart phones has become part of everyday life and a tool that helps with engaging with healthcare services and maintaining personal health.

As pointed out by Shakespeare et al.^79^ it must be noted that the frequency effect size used in meta‐summaries reflects the prevalence of a concept in the available literature, and not its significance or clinical relevance. For instance, two reports, published by the same research group, Johnsen et al.^32^, ^54^ were based on the same MAMAACT intervention in Denmark. Both were qualitative studies and discussed how women often sought medical advice from friends and family members. Therefore, the frequency effect size can be influenced by multiple reports being derived from the same intervention and does not accurately depict how insightful a concept is.

The role of personal health literacy is investigated in this review. However, the scope of this review does not include the role of organizations, despite their responsibility to facilitate access to health information. Therefore, further research on this subject is warranted as public health teams frequently aim to improve organizational health literacy.

Overall, further research is needed to investigate ways in which stillbirth could be reduced by increasing health literacy in socially disadvantaged pregnant women using smartphone‐based information. A recent example of such an effort is the DAiSI (Digital Animation in Service Improvement) project, a collection of digital animations available in English, Arabic and Urdu, relaying information about modifiable health behaviors to reduce adverse pregnancy outcomes.^80^ This project aims to increase health literacy and reduce language barriers by rendering the animations culturally sensitive and more accessible, without the need for reading in a given language.

In conclusion, this scoping review evaluated the availability of current literature on the topic of personal health literacy and its impact on stillbirth prevention in women of low socioeconomic status or ethnic minority background. The meta‐summary revealed associations between reduced health literacy, difficulties with interacting with health services, and the resulting potentially increased stillbirth risk. This phenomenon could be attributed to low health knowledge, unfamiliarity with health services, language barriers, illiteracy, and obtaining information from non‐official sources. However, the meta‐analysis demonstrated no significant reduction in stillbirth, maternal mortality and neonatal mortality after increased health knowledge through current interventions, which focus on participatory women's groups. Hence, descriptive studies have outlined the problems, but current interventions do not reduce the associated adverse outcomes. Further research must be done on the role of organizational health literacy and e‐literacy. Additionally, health literacy regarding fetal well‐being, particularly in lower‐resource settings, must be further researched, along with the currently ongoing efforts to improve maternal and infant well‐being. To facilitate interaction with health services and information, consistent provision of interpretation services and accessible health promotion tools is needed.

## AUTHOR CONTRIBUTIONS

The idea for this review was produced by AH and KW. JK conducted the database search, and JK and MW completed the screening and selection of studies. All authors contributed to the analysis. The initial version of the manuscript was produced by JK. JK, AH, KW, MW, and TS reviewed the manuscript and contributed to the final version.

## FUNDING INFORMATION

AH receives funding from Tommys Baby Charity.

## CONFLICT OF INTEREST STATEMENT

The authors have no conflicts of interest.

## Supporting information


Table S1.

Table S2.

Table S3.

Table S4.

Table S5.

Table S6.

Table S7.

## Data Availability

The data that support the findings of this study are available from the corresponding author upon reasonable request.
