# Supplementary material for: Impact of health literacy on pregnancy outcomes in socioeconomically disadvantaged and ethnic minority populations: A scoping review
Source: Int J Gynaecol Obstet. 2024 Aug 22;168(1):69–81. doi: 10.1002/ijgo.15852 (PMC11649848; doi:10.1002/ijgo.15852)
Supplement: Supplementary file 2 — Table S2. [file IJGO-168-69-s005.docx]

Table S2. Search strategies for each database

| MEDLINE | ((health literac* or health knowledge* or health attitude* or health awareness* or healthcare utili?ation* or healthcare seeking behavio?r* or information seeking behavio?r* or health communication* or communication*).ti,ab. **OR** (exp health education/ or exp consumer health information/ or exp health literacy/ or exp patient education as topic/ or exp prenatal education/ or exp attitude to health/ or exp health knowledge, attitudes, practice/ or exp "treatment adherence and compliance"/ or exp "patient acceptance of health care"/ or exp patient compliance/ or exp patient dropouts/ or exp patient participation/ or exp health behavior/ or exp health risk behaviors/ or exp information seeking behavior/ or exp risk reduction behavior/ or exp access to information/ or exp computer literacy/ or exp health communication/ or exp information dissemination/ or exp information literacy/ or exp internet access/ or exp communication/ or exp "cell phone use"/ or exp language/ or exp literacy/ or exp narration/ or exp narrative medicine/ or exp negotiating/ or exp teach-back communication/ or exp verbal behavior/))  **AND**  ((stillb*).ti,ab. **OR** (exp Stillbirth/))  **AND**  ((low socioeconomic or disadvantaged or underprivilege* or homeless* or impoverished or low income* or un?educat* or illiterate or unemploy* or jobless* or low living standard* or ethnic minorit* or racial minorit* or migrant* or foreigner* or national minorit*).ti,ab. **OR** (exp low socioeconomic status/ or exp Vulnerable Populations/ or exp socioeconomic factors/ or exp poverty/ or exp poverty areas/ or exp Ill-Housed Persons/ or exp Homeless Youth/ or exp educational status/ or exp academic failure/ or exp Unemployment/ or exp "ethnic and racial minorities"/ or exp minority groups/ or exp social marginalization/ or exp social vulnerability/ or exp "Transients and Migrants"/ or exp "emigrants and immigrants"/ or exp undocumented immigrants/)) |
| --- | --- |
| Cochrane Database of Systematic Reviews | ((health literac* OR health knowledge* OR health attitude* OR health awareness* OR healthcare utili?ation* OR healthcare seeking behavio?r* OR Information seeking behavio?r* OR Health communication* OR Communication):ti,ab,kw **OR** ((MeSH Health Literacy) OR (MeSH Consumer Health Information) OR (MeSH Health Education) OR (MeSH Patient Education as Topic) OR (MeSH Prenatal Education) OR (MeSH Health Knowledge, Attitudes, Practice) OR (MeSH Attitude to Health) OR (MeSH Treatment Adherence and Compliance) OR (MeSH Patient Acceptance of Health Care) OR (MeSH Patient Compliance) OR (MeSH Patient Participation) OR (MeSH Information Seeking Behavior) OR (MeSH Health Behavior) OR (MeSH Health Risk Behaviors) OR (MeSH Help-Seeking Behavior) OR (MeSH Health Communication) OR (MeSH Access to Information) OR (MeSH Communication Barriers) OR (MeSH Limited English Proficiency) OR (MeSH Computer Literacy) OR (MeSH Information Dissemination) OR (MeSH Information Literacy) OR (MeSH Information Literacy) OR (MeSH Internet Access) OR (MeSH Communication) OR (MeSH Cell Phone Use) OR (MeSH Language) OR (MeSH Literacy) OR (MeSH Negotiating)OR (MeSH Nonverbal Communication) OR (MeSH Verbal Behavior)))    **AND**    ((stillb*):ti,ab,kw **OR** (MeSH Stillbirth))    **AND**    ((Low socioeconomic OR Disadvantaged OR Underprivilege* OR Homeless* OR Impoverished OR Low income OR Un?educat* OR Illiterate OR Unemploy* OR Jobless* OR Low living standard* OR Ethnic minorit* OR Racial minorit* OR Migrant* OR Foreigner* OR National minorit*):ti,ab,kw **OR** ((MeSH Socioeconomic Factors) OR (MeSH Poverty) OR (MeSH ) OR (MeSH Poverty Areas) OR (MeSH Vulnerable Populations) OR (MeSH Ill-Housed Persons) OR (MeSH Homeless Youth) OR (MeSH Academic Performance) OR (MeSH Unemployment) OR (MeSH Ethnic and Racial Minorities) OR (MeSH Minority Groups) OR (MeSH Social Marginalization) OR (MeSH ) OR (MeSH Social Vulnerability) OR (MeSH Transients and Migrants) OR (MeSH Emigrants and Immigrants) OR (MeSH Undocumented Immigrants))) |
| CINAHL | (((TI health literac* OR AB health literac*) OR (TI health knowledge* OR AB health knowledge*) OR (TI health attitude* OR AB health attitude*) OR (TI health awareness* OR AB health awareness*) OR (TI healthcare utili?ation* OR AB healthcare utili?ation*) OR (TI healthcare seeking behavio?r* OR AB healthcare seeking behavio?r*) OR (TI information seeking behavio?r* OR AB information seeking behavio?r*) OR (TI health communication* OR AB health communication*) OR (TI communication* OR AB communication*)) **OR** ((MH "Health Literacy") OR (MH "Information Literacy+") OR (MH "Literacy") OR (MH "Computer Literacy") OR (MH "Consumer Health Information+") OR (MH "Health Literacy") OR (MH "Information Literacy+") OR (MH "Health Education+") OR (MH "School Health Education") OR (MH "Student Health Education") OR (MH "Patient Education+") OR (MH "Health Knowledge") OR (MH "Attitude to Medical Treatment") OR (MH "Attitude to Health+") OR (MH "Health Beliefs") OR (MH "Patient Compliance+") OR (MH "Medication Compliance") OR (MH "Attitude to Illness+") OR (MH "Attitude to Pregnancy") OR (MH "Attitude to Risk") OR (MH "Patient Attitudes") OR (MH "Patient Preference") OR (MH "Health Behavior (Iowa NOC)+") OR (MH "Adherence Behavior (Iowa NOC)") OR (MH "Compliance Behavior (Iowa NOC)") OR (MH "Health Promoting Behavior (Iowa NOC)") OR (MH "Health Seeking Behavior (Iowa NOC)") OR (MH "Participation: Health Care Decisions (Iowa NOC)") OR (MH "Symptom Control Behavior (Iowa NOC)") OR (MH "Health Behavior+") OR (MH "Help Seeking Behavior") OR (MH "Information Seeking Behavior") OR (MH "Risk Taking Behavior+") OR (MH "Health Seeking Behavior Alteration (Saba CCC)") OR (MH "Health Knowledge and Behavior (Iowa NOC)+") OR (MH "Internet Access") OR (MH "Language+") OR (MH "Negotiation") OR (MH "Nonverbal Communication+") OR (MH "Communication Skills") OR (MH "Verbal Behavior+") OR (MH "Access to Information+")  OR (MH "Computer Literacy") OR (MH "Communication+") OR (MH "Literacy")))    **AND**    ((TI stillb* OR AB stillb*) **OR** (MH "Perinatal Death"))    **AND**    (((TI low socioeconomic OR AB low socioeconomic) OR (TI disadvantaged OR AB disadvantaged) OR (TI underprivilege* OR AB underprivilege*) OR (TI homeless* OR AB homeless*) OR (TI impoverished OR AB impoverished) OR (TI low income OR AB low income) OR (TI un?educat* OR AB un?educat*) OR (TI illiterate OR AB illiterate) OR (TI unemploy* OR AB unemploy*) OR (TI jobless* OR AB jobless*) OR (TI low living standard* OR AB low living standard*) OR (TI ethic minorit* OR AB ethnic minorit*) OR (TI racial minorit* OR AB racial minorit*) OR (TI migrant* OR AB migrant*) OR (TI foreigner* OR AB foreigner*) OR (TI national minorit* OR AB national minorit*)) **OR** (MH "Minority Groups") OR (MH "Socioeconomic Factors+") OR (MH "Economic Status") OR (MH "Housing Instability") OR (MH "Poverty+") OR (MH "Social Class+") OR ((MH "Educational Status") OR (MH "Employment Termination") OR (MH "Poverty Areas") OR (MH "Unemployment") OR (MH "Homeless Persons") OR (MH "Public Housing") OR (MH "Academic Failure") OR (MH "Academic Performance+") OR (MH "Minority Groups") OR (MH "Ethnic Groups+") OR (MH "Immigrants+") OR (MH "Transients and Migrants") OR (MH "Undocumented Immigrants"))) |
| PsychINFO | ((health literac* or health knowledge* or health attitude* or health awareness* or healthcare utili?ation* or healthcare seeking behavio?r* or information seeking behavio?r* or health communication* or communication*).ti,ab. **OR** (exp health education/ or exp consumer health information/ or exp health literacy/ or exp patient education as topic/ or exp prenatal education/ or exp attitude to health/ or exp health knowledge, attitudes, practice/ or exp "treatment adherence and compliance"/ or exp "patient acceptance of health care"/ or exp patient compliance/ or exp patient dropouts/ or exp patient participation/ or exp health behavior/ or exp health risk behaviors/ or exp information seeking behavior/ or exp risk reduction behavior/ or exp access to information/ or exp computer literacy/ or exp health communication/ or exp information dissemination/ or exp information literacy/ or exp internet access/ or exp communication/ or exp "cell phone use"/ or exp language/ or exp literacy/ or exp narration/ or exp narrative medicine/ or exp negotiating/ or exp teach-back communication/ or exp verbal behavior/))  **AND**  ((stillb*).ti,ab. **OR** (exp Stillbirth/))  **AND**  ((low socioeconomic or disadvantaged or underprivilege* or homeless* or impoverished or low income* or un?educat* or illiterate or unemploy* or jobless* or low living standard* or ethnic minorit* or racial minorit* or migrant* or foreigner* or national minorit*).ti,ab. **OR** (exp low socioeconomic status/ or exp Vulnerable Populations/ or exp socioeconomic factors/ or exp poverty/ or exp poverty areas/ or exp Ill-Housed Persons/ or exp Homeless Youth/ or exp educational status/ or exp academic failure/ or exp Unemployment/ or exp "ethnic and racial minorities"/ or exp minority groups/ or exp social marginalization/ or exp social vulnerability/ or exp "Transients and Migrants"/ or exp "emigrants and immigrants"/ or exp undocumented immigrants/)) |
| LILACS | (tw:(health literac* OR health knowledge* OR health attitude* OR health awareness* OR healthcare utili?ation* OR healthcare seeking behavio?r* OR Information seeking behavio?r* OR Health communication* OR Communication) **OR** mh:(Attitude of Health Personnel OR Attitude to Death OR Attitude to Health OR Health Knowledge, Attitudes, Practice OR Treatment Adherence and Compliance OR Communication OR Health Behavior OR Illness Behavior OR Information Seeking Behavior OR Risk Reduction Behavior OR Risk-Taking OR Access to Information OR Communication Barriers OR Computer Literacy OR Health Communication OR Information Literacy OR Information Seeking Behavior OR Nonverbal Communication OR Language))  **AND**  (tw:(stillb*) **OR** mh:(stillbirth))  **AND**  (tw:(low socioeconomic or disadvantaged or underprivilege* or homeless* or impoverished or low income* or un?educat* or illiterate or unemploy* or jobless* or low living standard* or ethnic minorit* or racial minorit* or migrant* or foreigner* or national minorit*) **OR** mh:(Emigrants and Immigrants OR Undocumented Immigrants OR Refugees OR Transients and Migrants OR Working Poor OR Ethnic and Racial Minorities OR Minority Groups OR Vulnerable Populations OR Economic Stability OR Housing Instability OR Economic Status OR Poverty OR Educational Status OR Academic Failure OR Literacy OR Unemployment OR Ill-Housed Persons OR Homeless Youth OR Social Vulnerability)) |
