# Supplementary material for: Impact of health literacy on pregnancy outcomes in socioeconomically disadvantaged and ethnic minority populations: A scoping review
Source: Int J Gynaecol Obstet. 2024 Aug 22;168(1):69–81. doi: 10.1002/ijgo.15852 (PMC11649848; doi:10.1002/ijgo.15852)
Supplement: Supplementary file 3 — Table S3. [file IJGO-168-69-s003.docx]

Table S3: Characteristics of included qualitative studies table.

| **Author** | **Title**  **(Year)** | **Context** | **Country setting** | **Sample size** | **Study type** | **Ethnicity or socioeconomic factor** | **Health literacy concept** | **Key findings relating to the scoping review** |
| --- | --- | --- | --- | --- | --- | --- | --- | --- |
| Ujwala Bapat et al. | Stillbirths and newborn deaths in slum settlements in Mumbai, India: a prospective verbal autopsy study^38^  (2012) | Perinatal death | India (lower-middle income) | n=13,467 births | Prospective verbal autopsy study | Slum areas  Education  Socioeconomic quintile | Knowledge and understanding of pregnancy-related information  Compliance | Poor recognition of symptoms or the severity of maternal complications contributed to delayed care-seeking amongst women in slum settlements in Mumbai.  Reasons for feeling responsible for the death included: (1) being careless with their own health during pregnancy; (2) failing to seek care during illness; (3) not following health advice.  Perinatal death not associated with education level but strongly associated with socio-economic status. |
| Andrea Blanchard et al. | Understanding the roles of community health workers in improving perinatal health equity in rural Uttar Pradesh, India: a qualitative study^44^  (2021) | Community health workers | India (lower-middle income) | n=134 participants | Focus groups | Socioeconomic factors (wealth, education, access to facilities, occupation, religion, and caste) | Knowledge and perception of health service | Informing women about affordable, professional care and the importance and benefit of institutional delivery contributed to an increase in institutional delivery. |
| Mary Carolan | Diabetes nurse educators’ experiences of providing care for women, with gestational diabetes mellitus, from disadvantaged backgrounds^40^(2014) | Gestational diabetes mellitus | Australia (high-income) | n= 6 diabetes nurse educators | Interviews | Socially disadvantaged  multi-ethnic population  Non-English-speaking background  Unemployed  Assisted housing  Socioeconomic status  Education level | Form of literacy  Knowledge and understanding of pregnancy-related information  Language barrier | Literacy hindered sharing of information with the patients.  There were beliefs regarding nutrition in which coconut milk and ghee were thought to lead to a fairer skinned baby.  Some women did not maintain a healthy diet during their pregnancy.  Exercise was deemed unsafe for the baby.  Language barrier made understanding the changes that had to be made more difficult for the women. |
| Aliki Christou et al. | Understanding pathways leading to stillbirth: The role of care-seeking and care received during pregnancy and childbirth in Kabul province, Afghanistan^11^  (2020) | Perinatal care | Afghanistan (low-income) | n= 55 participants | Semi - structured interviews | Poor socioeconomic status  Inability to afford medications, treatment, and antenatal care | Knowledge and perception of health service  Knowledge and understanding of pregnancy-related information | Some with history of uneventful pregnancies believed care was unnecessary.  ANC was generally deemed unimportant.  There was a general lack of awareness regarding what to do and what not to do during pregnancy, and many women participated in strenuous physical work.  Knowledge of nutrition was poor.  Knowledge of birth spacing was poor, leading to increased risk of anemia.  Due to lack of awareness regarding risks of certain medications during pregnancy, many continued to take their usual medications.  Many refused surgical interventions due to their fear of surgery causing infertility and their belief that recovery time would be longer. |
| Julia Funge et al. | “No Papers. No Doctor”: A Qualitative Study of Access to Maternity Care Services for Undocumented Immigrant Women in Denmark^45^  (2020) | Maternity care services | Denmark (high-income) | n= 21 women | semi-structured interviews | Undocumented immigrant women | Knowledge and perception of health service  Obtaining information | Some undocumented immigrant women were unsure about the care they could receive.  Friends and family were the women's main source of information and comfort.  Another source of information was the internet due to its easily accessible nature.  They were aware the internet was not completely trustworthy. |
| Rebecca Garcia et al. | A qualitative study exploring the experiences of bereavement after stillbirth in Pakistani, Bangladeshi, and white British mothers living in Luton, UK^39^  (2020) | Bereavement | United Kingdom (high-income) | n= 6 women | Semi - structured interviews | Ethnicities (Pakistani, Bangladeshi, and White British mothers) | Knowledge and understanding of pregnancy-related information  Knowledge and perception of health service | Regardless of ethnicity, all mothers believed that they had limited knowledge of stillbirth prevention.  There were beliefs around avoiding certain foods for the health of the mother and the fetus.  Several mothers believed that the fetal anomaly scan was harmful towards the fetus and rejected the scan. |
| Ramprakash Kaswa et al. | Exploring the pregnant women's perspective of late booking of antenatal care services at Mbekweni Health Centre in Eastern Cape, South Africa.^8^  (2018) | Antenatal care | South Africa (upper-middle income) | n= 20 pregnant women | Semi - structured interviews | Affordability | Obtaining information  Language barrier | The main source of information regarding ANC was mothers or peers instead of healthcare professionals.  Language barrier contributed to delayed ANC attendance in women with occupations in other provinces.  Not knowing the location of the ANC facility contributed to delayed ANC attendance in women with occupations in other provinces |
| Elizabeth Krans et al. | Low-Income African American Women’s Beliefs Regarding Exercise during Pregnancy^33^  (2011) | Exercise during pregnancy | United States (high-income) | n=34 women | Focus groups | African American  Annual household income  Education completed | Knowledge and understanding of pregnancy-related information  Obtaining information | Participants had a broad definition of exercise, considering activities of daily living as exercise.  They deemed exercise as having benefits during pregnancy.  Some believed that overexertion and specific movements could be detrimental to the fetus.  Some lacked knowledge regarding the parameters of safe exercise (e.g. frequency, type, duration).  Many obtained information regarding the effect of exercise on labor and delivery from family members and partners. |
| Helle Johnsen et al. | Implementing the MAMAACT intervention in Danish antenatal care: a qualitative study of non-Western immigrant women's and midwives’ attitudes and experiences^47^  (2021) | Antenatal care | Denmark  (high-income) | n= 71 midwives and non-Western immigrant women | Multi - method qualitative study | Non-Western immigrant women | Knowledge and understanding of pregnancy-related information  Language barrier  Form of literacy | The leaflet and app provided women with relevant information on potential pregnancy complications and how to act.  Due to their availabilities in various languages, language barrier was reduced and access to information was facilitated.  The smartphone app was not easily accessible to some due to low e-health literacy. |
| Helle Johnsen et al. | Contextual Factors Influencing the MAMAACT Intervention: A Qualitative Study of Non-Western Immigrant Women’s Response to Potential Pregnancy Complications in Everyday Life^54^  (2020) | Response to pregnancy complication | Denmark (high-income) | n=21 non-Western immigrant women | Semi-structured interviews | Maternal education level  Non-Western immigrant women | Obtaining information | Women sought advice from female friends as they were deemed trustworthy.  Sometimes this advice became a barrier for seeking medical advice.  The internet was a source of information as well as they perceived online material to be trustworthy. However, they rarely utilized hospital websites for obtaining information. |
| Helle Johnsen et al. | Addressing ethnic disparity in antenatal care: a qualitative evaluation of midwives’ experiences with the MAMAACT intervention  (2020)^32^ | Antenatal care | Denmark (high-income) | n=18 midwives | Mini-group interviews | Non-Western immigrant women | Obtaining information  Knowledge and understanding of pregnancy-related information  Language barrier  Knowledge and perception of health service | Non-Western immigrant women were more likely to obtain information from relatives instead of midwives. The information they obtained was not always correct.  There was a lack of knowledge regarding physiology and anatomy and concern that this lack of awareness could affect their timely response to pregnancy symptoms.  The MAMAACT leaflet was perceived positively by the midwives as they believed the leaflet could aid immigrant women who have recently arrived with navigating antenatal care and gaining information about pregnancy symptoms.  Language proficiency was reported to be an issue amongst many non-Western immigrants when communicating with health professionals. |
| Tomasina Stacey et al. | An exploration of migrant women’s perceptions of public health messages to reduce stillbirth in the UK: a qualitative study^14^  (2021) | Public health messages during pregnancy | United Kingdom (high-income) | n=30 migrant women | Semi - structured focus groups  Interviews | Migrant women | Knowledge and understanding of pregnancy-related information  Obtaining information  Form of literacy | Migrant women had poor understanding of stillbirth, both in English and in their mother tongue.  Although generally knowledgeable about key messages regarding keeping their babies safe, some were not aware that these advice were beneficial for reducing stillbirth.  Women received varying advice regarding daily life, including diet, from friends and family and health professionals. This variation and inconsistency led to confusion.  Information from health professionals was deemed the most reliable.  Internet was a major source of information. The NHS website was frequently used yet some found the information too general.  Some women did not think written information would be useful as many lacked literacy. Some believed that low E-literacy would hinder women from utilizing smartphone apps for accessing information. |
