# Supplementary material for: Impact of health literacy on pregnancy outcomes in socioeconomically disadvantaged and ethnic minority populations: A scoping review
Source: Int J Gynaecol Obstet. 2024 Aug 22;168(1):69–81. doi: 10.1002/ijgo.15852 (PMC11649848; doi:10.1002/ijgo.15852)
Supplement: Supplementary file 4 — Table S4. [file IJGO-168-69-s004.docx]

Table S4: Characteristics of included descriptive survey studies table.

| **Author** | **Title**  **(Year)** | **Context** | **Country setting** | **Sample size** | **Study type** | **Ethnicity or socioeconomic factor** | **Health literacy concept** | **Key findings relating to the scoping review** |
| --- | --- | --- | --- | --- | --- | --- | --- | --- |
| Abdirasak Sharif Ali et al. | Seroprevalence of Syphilis among Pregnant Women Attending Antenatal Care in Yaqshiid District, Mogadishu, Somalia^43^ (2024) | Syphilis | Somalia (low-income) | n = 300 pregnant women | cross-sectional study | Employment, education, residence | Knowledge and understanding of pregnancy-related information | Majority of the participants lacked employment (69%) and formal education (73%). Majority of the participants also lacked knowledge regarding syphilis-related information. However, lacking employment, formal education and good knowledge of syphilis were associated with reduced prevalence of syphilis infection. |
| Noon Altijani et al. | Stillbirth among women in nine states in India: rate and risk factors in study of 886,505 women from the annual health survey^55^  (2018) | risk factors | India (lower-middle-income) | n=886,505 women | survey | Low socioeconomic status  Schedule caste background | Form of literacy | 84.8% of the women only had primary and below education or were illiterate and 83.6% were not in paid employment. 31.6% were in a schedule caste or tribe group and 35.9% were in the lowest or second to lowest asset index quintile. Female illiteracy, belonging to a schedule caste background and low socioeconomic status were significantly associated with higher stillbirth rate. |
| Verena Carrara et al. | Improved pregnancy outcome in refugees and migrants despite low literacy on the Thai-Burmese border: results of three cross-sectional surveys^57^  (2011) | risk of stillbirth | Thailand (upper-middle income) | n=2424 pregnant women | Cross-sectional survey | Migrant  Refugee | Form of literacy | Literacy was associated with the following demographic characteristics: delivery at health facility, non-smoking and malaria-free during pregnancy. |
| Samira Behboudi-Gandevani et al. | Iranian primigravid women's awareness of the risks associated with delayed childbearing.^36^(2013) | Risk awareness | Iran (lower-middle income) | n=675 primigravid pregnant women | Cross-sectional survey | Education level  Employment status  Family income | Knowledge and understanding of pregnancy-related information | Education level was strongly associated with awareness of risks of childbearing (e.g. stillbirth risk) in advanced maternal age as those with lower levels of education (≤ high school graduate) had poorer knowledge of maternal age-related risks of childbearing (OR 2.01, 95% CI 1.72-3.45). |
| Mary Carolan et al. | Attitudes towards gestational diabetes among a multiethnic cohort in Australia^37^  (2010) | Gestational diabetes | Australia (high-income) | n= 143 women with gestation diabetes mellitus | cross-sectional survey | Maternal education  Ethnicity | Knowledge and understanding of pregnancy-related information | There was an association between lower education level and awareness of the danger of gestational diabetes and the value of tight glycemic control. |
| Manal Abdalla Sayed Ahmed Gaheen et al. | Knowledge and attitude of women regarding toxoplasmosis during pregnancy and measures to overcome it in slum areas^34^  (2014) | toxoplasmosis prevention | Egypt (lower-middle-income) | n= 302 women | Descriptive design | Slum area  Maternal education | Knowledge and understanding of pregnancy-related information  Obtaining information | Participants were from slum areas. 79.8% of the participants had secondary level of education. 69.4% of the participants had poor knowledge of toxoplasmosis. The knowledge score was significantly higher in women with university education.  Friends and family, followed by neighbors were the main source of information regarding toxoplasmosis (46.69% and 33.44%, respectively). |
| Jaameeta Kurji et al. | Factors associated with maternity waiting home use among women in Jimma Zone, Ethiopia: a multilevel cross-sectional analysis^46^  (2019) | Maternity waiting home | Ethiopia (low-income) | n= 3784 women with pregnancy outcome | cross-sectional analysis of baseline household survey data | Household wealth  Education levels | Knowledge and perception of health service | Although 71% of women interviewed knew of the MWH service and most were aware of at least one benefit of the service, they lacked a comprehensive understanding of the service and only 7% of the women had previously used MWH services.  Wealthier households were statistically more likely to use MWH services, while education was not associated with MWH use. |
| Jesmin Pervin et al. | Level and determinants of birth preparedness and complication readiness among pregnant women: A cross sectional study in a rural area in Bangladesh^41^  (2018) | birth preparedness and complication readiness | Bangladesh (lower-middle income) | n=2262 women | cross-sectional survey | Level of education  Asset index (1=poorest; 5=richest) | Knowledge and understanding of pregnancy-related information | 33.2% and 24.3% of the women could not mention any danger signs for pregnancy and delivery, respectively.  Having a good knowledge of maternal danger signs was associated with increased preparedness. |
| Mohammad Suliman et al. | Toxoplasmosis prevention: knowledge and practices among pregnant women in Jordan^35^  (2021) | Toxoplasmosis prevention | Jordan (upper-middle-income) | n = 583 pregnant women | cross-sectional descriptive study | Employment status  Income level  Nationality | Knowledge and understanding of pregnancy-related information | Pregnant women's knowledge of toxoplasmosis was not affected by employment status or income level. The level of knowledge was significantly lower amongst Syrian women compared to Jordanian women (OR=1.99). |
