# Supplementary material for: Impact of health literacy on pregnancy outcomes in socioeconomically disadvantaged and ethnic minority populations: A scoping review
Source: Int J Gynaecol Obstet. 2024 Aug 22;168(1):69–81. doi: 10.1002/ijgo.15852 (PMC11649848; doi:10.1002/ijgo.15852)
Supplement: Supplementary file 5 — Table S5. [file IJGO-168-69-s006.docx]

Table S5: Characteristics of included observational analytic studies table.

| **Author** | **Title**  **(Year)** | **Context** | **Country setting** | **Sample size** | **Study type** | **Ethnicity or socioeconomic factor** | **Health literacy concept** | **Key findings relating to the scoping review** |
| --- | --- | --- | --- | --- | --- | --- | --- | --- |
| Andreea A. Creanga et al. | Pregnant Women's Intentions and Subsequent Behaviors Regarding Maternal and Neonatal Service Utilization: Results from a Cohort Study in Nyanza Province, Kenya.^69^  (2016) | Maternal and neonatal service | Kenya (lower-middle-income) | n= 1,056 pregnant women | Prospective cohort study | Education level  Inability to afford healthcare services | Knowledge and perception of health service | 22 out of 1,056 women experienced stillbirth and secondary education was completed in 1 out of 5 women. Intention to utilize ANC and delivery at a healthcare facility were high (98.8%-99.4% and 89.0%-96.2%, respectively). Actual service use was 98.1% and 76.9%. Having 9+ years of education compared to 5-8 years was one of the predictors of discordance between intention and actual place of delivery. Inability to afford health services was a reason for not delivering at their "preferred" place. |
| Fiona Cross-Sudworth et al. | Perinatal deaths of migrant mothers: Adverse outcomes from unrecognized risks and substandard care factors.^50^  (2015) | System failure | United Kingdom (high-income) | n= 36 perinatal deaths | Retrospective case-control | Migrant women  Social risk factors | Language barrier | In 83% of cases in which the mother did not speak English, professional translation services were not available to them. |
| Sven g. Hinderaker et al. | Avoidable stillbirths and neonatal deaths in rural Tanzania^42^ (2003) | Risk factors for stillbirth | Tanzania (lower-middle income) | n=139 registered deaths | Prospective cohort study and retrospective survey | Low-income setting | Knowledge and understanding of pregnancy-related information  Knowledge and perception of health service | Risk factors for fetal mortality were present in 11 of the 13 avoidable stillbirths. However, only 1 mother was aware of the risk factor and all 13 mothers claimed they were not referred for the risk factor |
| Maryam Mozooni et al. | Healthcare factors associated with the risk of antepartum and intrapartum stillbirth in migrants in Western Australia (2005-2013): A retrospective cohort study^48^  (2020) | risk of stillbirth | Australia (high-income) | n= 260,997 total births | retrospective cohort study | Migrant women | Language barrier | Interpreter service utilization in migrant women was associated with lower rate of stillbirth (0.26% vs 0.58%). This was especially evident among African migrant women (0.31% vs 1.41%) |
| Maryam MozooniI, et al. | The influence of acculturation on the risk of stillbirth in migrant women residing in Western Australia^49^  (2020) | risk of stillbirth | Australia (high-income) | n= 260,997 total births | retrospective cohort study | Migrant women | Language barrier | Highest use of interpretation services was found in African migrant women. Usage of an interpreter was associated with lower odds of stillbirth, compared to when an interpreter was not used, in non-white-non-maori women (OR 0.47 vs OR 1.55) |
| A Sepou et al. | [Prenatal care in a semiurban area of Central African Republic: frequency, influential factors, maternal and neonatal prognosis]^31^  (2000) | Antenatal care | Central African Republic (low-income) | n=287 women | Longitudinal cohort study | Inability to afford ANC | Form of literacy  Knowledge and perception of health service | There was an association between illiteracy and poor ANC attendance. Reasons for not attending ANC included perceiving ANC as a service that is useless and not being able to afford the service. |
| Irene Sterpu et al. | Could a multidisciplinary regional audit identify avoidable factors and delays that contribute to stillbirths? A retrospective cohort study^51^  (2020) | risk factors | Sweden (high-income) | n=28,805 deliveries | retrospective cohort study | Country of birth | Language barrier | Stillbirth outcomes were divided into possibly preventable, probably preventable, and non-preventable.  The country of birth of the mother (in/outside of Sweden) was not associated with significant changes in the probably preventable stillbirth outcome: 14% vs 17% (p=0.36).  However, the differences of possibly preventable, probably preventable, and non-preventable stillbirth outcome in women who spoke Swedish and those who did not were statistically significant (p=0.03).  Additionally, not speaking Swedish was associated with increased patient-associated delay compared to those who spoke Swedish (37% vs 11%, p=0.02)" |
| Daniel Tarekegn Worede et al. | Determinants of stillbirth in Felege-Hiwot comprehensive specialized referral hospital, North-west, Ethiopia, 2019^56^  (2019) | specialized comprehensive referral hospital | Ethiopia (low-income) | n= 420 post-partum women | case-control study | Education level | Form of literacy | The proportion of mothers who received tertiary education was greater in the control group (live birth outcome) compared to the case group (stillbirth outcome) (28.3% vs 11.9%). It was presumed that lack of education results in illiteracy.  women who were illiterate (AOR=3.8, 95% CI 1.4–10.2) had high odds of stillbirth compared to women had tertiary education. |
