# Supplementary material for: Impact of health literacy on pregnancy outcomes in socioeconomically disadvantaged and ethnic minority populations: A scoping review
Source: Int J Gynaecol Obstet. 2024 Aug 22;168(1):69–81. doi: 10.1002/ijgo.15852 (PMC11649848; doi:10.1002/ijgo.15852)
Supplement: Supplementary file 6 — Table S6. [file IJGO-168-69-s001.docx]

Table S6: Characteristics of included intervention studies table.

| **Author** | **Title**  **(Year)** | **Context** | **Country setting** | **Sample size** | **Study type** | **Ethnicity or socioeconomic factor** | **Health literacy concept** | **Key findings relating to the scoping review** |
| --- | --- | --- | --- | --- | --- | --- | --- | --- |
| Kishwar Azad et al. | Effect of scaling up women’s groups on birth outcomes in three rural districts in Bangladesh: a cluster-randomized controlled trial^63^  (2010) | Women's group | Bangladesh (lower-middle-income) | n= 36,113 births monitored | cluster-randomized controlled trial | Maternal education  Access to perinatal health care | Health education | Intervention: women’s group in which women were improving their knowledge on perinatal experiences through discussions and taking relevant local actions.   \|  \| Control \| Intervention \| \| --- \| --- \| --- \| \| Births \| 15257 \| 15695 \| \| Stillbirth \| 521 \| 542 \| \| Neonatal mortality \| 557 \| 515 \| \| Maternal mortality \| 55 \| 32 \| |
| Tim Colbourn et al. | Effects of quality improvement in health facilities and community mobilization through women's groups on maternal, neonatal and perinatal mortality in three districts of Malawi: MaiKhanda, a cluster randomized controlled effectiveness trial^66^  (2016) | Women’s group | Malawi  (low-incme) | n=20,576 births | Cluster-randomized controlled trial | Social causes of maternal death (illiteracy, poverty, low knowledge) | Health education | Intervention: women’s group   \|  \| Control \| Intervention \| \| --- \| --- \| --- \| \| Births \| 4912 \| 10329 \| \| Stillbirth \| 146 \| 274 \| \| Neonatal mortality \| 162 \| 286 \| \| Maternal mortality \| 10 \| 22 \| |
| Edward Fottrell et al. | The Effect of Increased Coverage of Participatory Women’s Groups on Neonatal Mortality in Bangladesh: A Cluster Randomized Trial^65^  (2016) | Women’s group | Bangladesh  (lower-middle-income) | n=19,301 births analyzed | Cluster-randomized controlled trial | Maternal education  Access to perinatal healthcare | Health education | Intervention: women’s group   \|  \| Control \| Intervention \| \| --- \| --- \| --- \| \| Births \| 8834 \| 9106 \| \| Stillbirth \| 232 \| 287 \| \| Neonatal mortality \| 271 \| 187 \| \| Maternal mortality \| 23 \| 14 \| |
| Sonia Odette Lewycka | Reducing maternal and neonatal deaths in rural Malawi : evaluating the impact of a community-based women's group intervention^60^  (2011) | Women's group | Malawi (low-income) | n= 18,562 pregnancies | cluster-randomized | Socioeconomic score | Health education | Intervention: women’s group   \|  \| Control \| Intervention \| \| --- \| --- \| --- \| \| Births \| 9537 \| 9165 \| \| Stillbirth \| 167 \| 195 \| \| Neonatal mortality \| 219 \| 215 \| \| Maternal mortality \| 39 \| 34 \| |
| Dharma S Manandhar et al. | Effect of a participatory intervention with women's groups on birth outcomes in Nepal: cluster-randomized controlled trial^61^  (2004) | Women's group | Nepal (lower-middle-income) | n= 28,931 women and their birth outcomes | cluster-randomized controlled trial | Household asset score  Household food sufficiency  Education | Health education | Intervention: women’s group   \|  \| Control \| Intervention \| \| --- \| --- \| --- \| \| Births \| 3303 \| 2972 \| \| Stillbirth \| 77 \| 73 \| \| Neonatal mortality \| 119 \| 76 \| \| Maternal mortality \| 11 \| 2 \| |
| Ammal M. Metwally et al. | Strengths of community and health facilities-based interventions in improving women and adolescents’ care seeking behaviors as approaches for reducing maternal mortality and improving birth outcome among low-income communities of Egypt^67^  (2020) | Educational intervention | Egypt (lower-middle income) | n= 20,494 women | Interventional evaluation study | Low-income communities | Health education | Intervention: teaching, distribution of educational materials and improving recognition of signs of pregnancy complications.  The studied areas had limited healthcare access.  A statistically significant increase in the uptake of ANC was seen after exposure to the intervention. The intervention led to a significant reduction in perinatal complications and stillbirths. |
| Neena Shah More et al. | Community Mobilization in Mumbai Slums to Improve Perinatal Care and Outcomes: A Cluster Randomized^58^  (2012) | Women's groups | India (lower-middle income) | n=18,197 births | cluster randomized controlled trial | slum areas | Health education | Intervention: women's group   \|  \| Control \| Intervention \| \| --- \| --- \| --- \| \| Births \| 9042 \| 9155 \| \| Stillbirth \| 85 \| 73 \| \| Neonatal mortality \| 88 \| 132 \| \| Maternal mortality \| 24 \| 20 \| |
| Trine Damsted Rasmussen et al. | Effectiveness evaluation of an antenatal care intervention addressing disparities to improve perinatal outcomes in Denmark: A nationwide register-based analysis of a cluster randomized controlled trial (MAMAACT)^59^  (2022) | Health education material | Denmark (high-income) | n = 188,658 births | Cluster randomized controlled trial | Immigrants (Western and non-western) | Health education | Intervention: MAMAACT intervention (leaflet and smartphone app in six languages for pregnant women to increase their awareness on complication signs and instructions on how to act.)   \|  \| Control \| Intervention \| \| --- \| --- \| --- \| \| Births \| 20040 \| 31133 \| \| Stillbirth \| 73 \| 108 \| \| Neonatal mortality \| 30 \| 50 \| |
| Trine Damsted Rasmussen et al. | Improving health literacy responsiveness to reduce ethnic and social disparity in stillbirth and infant health: a cluster randomized controlled effectiveness trial of the MAMAACT intervention^64^  (2023) | Health education material | Denmark (high-income) | n= 4,150 women | cluster-randomized controlled trial | Immigrant women | Health education | Intervention: MAMAACT intervention  The intervention did not result in a statistically significant change in the non-Western immigrant participants' active engagement with healthcare providers, their ability to navigate the healthcare system or their certainty regarding complication management. |
| Prasanta Tripathy et al. | Effect of a participatory intervention with women’s groups on birth outcomes and maternal depression in Jharkhand and Orissa, India: a cluster-randomized controlled trial^62^  (2010) | Women's group | India (lower-middle-income) | n= 19,030 births monitored | cluster-randomized controlled trial | Household asset  Caste/tribal group  Maternal education | Health education | Intervention: women’s groups   \|  \| Control \| Intervention \| \| --- \| --- \| --- \| \| Births \| 9089 \| 9686 \| \| Stillbirth \| 270 \| 298 \| \| Neonatal mortality \| 518 \| 397 \| \| Maternal mortality \| 60 \| 49 \| |
