# Supplementary material for: Impact of health literacy on pregnancy outcomes in socioeconomically disadvantaged and ethnic minority populations: A scoping review
Source: Int J Gynaecol Obstet. 2024 Aug 22;168(1):69–81. doi: 10.1002/ijgo.15852 (PMC11649848; doi:10.1002/ijgo.15852)
Supplement: Supplementary file 7 — Table S7. [file IJGO-168-69-s002.docx]

Table S7: Characteristics of included perinatal audit studies table.

| **Author** | **Title**  **(year)** | **Context** | **Country setting** | **Sample size** | **Type of study** | **Ethnicity or socioeconomic factor** | **Health literacy concept** | **Key findings relating to the scoping review** |
| --- | --- | --- | --- | --- | --- | --- | --- | --- |
| Birgitta Essén et al. | Are some perinatal deaths in immigrant groups linked to suboptimal perinatal care services?^52^  (2002) | Perinatal care services | Sweden (high-income) | N=189 perinatal deaths | Audit | Women from Ethiopia, Somalia, or Eritrea (ESE) | Language barrier | Out of the 62 perinatal deaths in the ESE group, 69% were stillbirths and 5 were due to verbal miscommunication and lack of interpreter present. In contrast, out of 113 perinatal deaths in the Swedish group, 65% were stillbirths and none of the perinatal deaths were due to miscommunication. |
| Eli Saastad et al. | Suboptimal care in stillbirths - a retrospective audit study^53^  (2007) | Perinatal care services | Norway (high-income) | N=356 stillbirths | Audit | Non-western women | Language barrier | Non-Western women had significantly increased stillbirth risk compared to Western women (OR: 2.2; 95% CI: 1.3 - 3.8) and significantly lower education level. Miscommunication between the patient and the caregiver was significantly greater in non-Western women (47% vs 1%; p<0.001). |
